# Prevalence of Trachoma in Pakistan: Results of 42 Population-Based Prevalence Surveys from the Global Trachoma Mapping Project

**DOI:** 10.1080/09286586.2019.1708120

**Published:** 2020-01-09

**Authors:** Asad Aslam Khan, Victor V. Florea, Arif Hussain, Zahid Jadoon, Sophie Boisson, Rebecca Willis, Michael Dejene, Ana Bakhtiari, Caleb Mpyet, Alexandre L. Pavluck, Munazza Gillani, Babar Qureshi, Anthony W. Solomon

**Affiliations:** a Ministry of National Health Services, Regulations and Coordination, Islamabad, Pakistan; b College of Ophthalmology and Allied Vision Sciences, King Edward Medical University, Lahore, Pakistan; c Mayo Hospital, Lahore, Pakistan; d Department of Control of Neglected Tropical Diseases, World Health Organization, Geneva, Switzerland; e Pakistan Institute of Community Ophthalmology, Hayatabad Medical Complex, Peshawar, Pakistan; f Department of Public Health, The Environment and Social Determinants of Health, World Health Organization, Geneva, Switzerland; g International Trachoma Initiative, Task Force for Global Health, Decatur, GA, USA; h Michael Dejene Public Health Consultancy Services, Addis Ababa, Ethiopia; i Department of Ophthalmology, University of Jos, Jos, Nigeria; j Sightsavers, Kaduna, Nigeria; k Kilimanjaro Centre for Community Ophthalmology International, Division of Ophthalmology, University of Cape Town, Cape Town, South Africa; l Sightsavers, Islamabad, Pakistan; m Neglected Tropical Diseases, CBM, Oakington, Cambridge, UK; n Eastern Mediterranean Region Alliance for Trachoma Control, Cairo, Egypt; o Clinical Research Department, London School of Hygiene & Tropical Medicine, London, UK; p London Centre for Neglected Tropical Disease Research, London, UK

**Keywords:** Pakistan, trachoma, trichiasis, Global Trachoma Mapping Project, water, sanitation

## Abstract

***Purpose***: Previous phases of trachoma mapping in Pakistan completed baseline surveys in 38 districts. To help guide national trachoma elimination planning, we set out to estimate trachoma prevalence in 43 suspected-endemic evaluation units (EUs) of 15 further districts.

***Methods***: We planned a population-based trachoma prevalence survey in each EU. Two-stage cluster sampling was employed, using the systems and approaches of the Global Trachoma Mapping Project. In each EU, residents aged ≥1 year living in 30 households in each of 26 villages were invited to be examined by trained, certified trachoma graders. Questionnaires and direct observation were used to evaluate household-level access to water and sanitation.

***Results***: One EU was not completed due to insecurity. Of the remaining 42, three EUs had trichiasis prevalence estimates in ≥15-year-olds ≥0.2%, and six (different) EUs had prevalence estimates of trachomatous inflammation—follicular (TF) in 1–9-year-olds ≥5%; each EU requires trichiasis and TF prevalence estimates below these thresholds to achieve elimination of trachoma as a public health problem. All six EUs with TF prevalences ≥5% were in Khyber Pakhtunkhwa Province. Household-level access to improved sanitation ranged by EU from 6% to 100%. Household-level access to an improved source of water for face and hand washing ranged by EU from 37% to 100%.

***Conclusion***: Trachoma was a public health problem in 21% (9/42) of the EUs. Because the current outbreak of extremely drug-resistant typhoid in Pakistan limits domestic use of azithromycin mass drug administration, other interventions against active trachoma should be considered here.

## Introduction

Trachoma is a neglected tropical disease^1^ and the leading infectious cause of blindness worldwide.^2^ Trachomatous inflammation— follicular (TF) and/or trachomatous inflammation—intense (TI) are signs of active trachoma that can occur when certain strains^3,4^ of the bacterium *Chlamydia trachomatis* infect the conjunctivae. Trichiasis is a complication of trachoma that occurs after multiple episodes of infection and associated inflammation,^5^ typically in adulthood.^6–8^ It is characterized by in-turned eyelashes touching the eyeball, which may lead to pain, corneal ulceration, corneal scarring, and visual impairment.^9–11^

To eliminate trachoma as a public health problem,^12^ the World Health Organization (WHO) recommends implementation of the SAFE strategy: Surgery to redirect in-turned eyelashes away from the eye; Antibiotics to clear infection; and Facial cleanliness and Environmental improvement to reduce transmission.^13,14^ In order to plan and implement SAFE interventions, population-based prevalence surveys of TF and trichiasis are needed in districts suspected to be trachoma-endemic.^15,16^ Public health-level implementation of S is indicated in districts where trachomatous trichiasis prevalence is ≥0.2% in persons aged ≥15 years, while public health-level implementation of A, F and E is indicated in districts where TF prevalence is ≥5% in children aged 1–9 years.^17^

In Pakistan in the 1950s, trachoma was believed to account for >60% of all blindness nationally.^18^ The country now has a population of over 200 million people, 63% of whom live in rural areas.^19^ In addition to its Federal Capital Territory (Islamabad), Pakistan is comprised of four provinces (Baluchistan, Khyber Pakhtunkhwa, Punjab and Sindh), two autonomous territories (Azad Jammu & Kashmir, and Gilgit-Baltistan) and the Federally Administered Tribal Area. These province-level units are further split into the successively lower administrative tiers of 34 divisions, 149 districts (zillahs), and 588 sub-districts (tehsils).

From 2002 to 2004, the National Trachoma Task Force of Pakistan oversaw Trachoma Rapid Assessments (TRAs)^20^ covering 233 villages in Baluchistan (49 TRAs), North-West Frontier Province [renamed Khyber Pakhtunkhwa in 2010] (69), Punjab (67), Sindh (23) and Northern Areas [renamed Gilgit-Baltistan in 2009] (25).^21^ Based on these TRAs, from 2011 to 2013, prevalence surveys were undertaken over three phases in 38 districts. In each of those districts, 70 villages were chosen with a probability-proportional-to-size sampling strategy. In every selected village, systematic sampling was used to select households to facilitate examination of 100 adults. In every second selected village, 100 children were also examined. This produced relatively large sample sizes of 7000 adults and 3500 children per district.^22^ In Punjab province, the districts of DG Khan (0.77%), Hafizabad (0.39%), Narowal (1.01%) and Vehari (0.36%) had trichiasis prevalences in adults ≥0.2%; elsewhere, only Chitral (Khyber Pakhtunkhwa, 1.3%), Pishin (Baluchistan, 0.47%) and Skardu (Gilgit Baltistan, 0.93%) exceeded this threshold.

Across all provinces, only the districts of Chitral (Khyber Pakhtunkhwa, 12.7%) and Shadadkot (Sindh, 9.9%), had active trachoma (TF and/or TI) prevalence in 1–9-year-olds of ≥5%. Access to clean water was noted to be a major problem in almost all districts.^22^^22.^The aim of the present work was to (1) estimate the prevalence of TF among children aged 1–9 years and of trichiasis among persons aged ≥15 years, and (2) determine the proportion of households with access to improved water and sanitation facilities, in selected areas of Pakistan. This formed part of the Global Trachoma Mapping Project (GTMP).^23^ This information was needed to direct trachoma elimination efforts at national, provincial and sub-district levels.

## Methods

Prior to this series of surveys, as mentioned above, trachoma surveys had been completed in 38 districts of Pakistan. The work described here was considered a fourth phase of surveys. It was completed in 2015 and was intended by the National Trachoma Task Force to complete baseline surveys in suspected-endemic areas which did not then have valid prevalence data. Such suspected-endemic-but-unmapped areas were found across 29 districts, incorporating an estimated total population of 40,720,310. Due to time limitations and insecurity in some areas, 14 of the 29 districts were excluded from the plan. In the remaining 15 districts, we deprioritized urban areas (where risk of trachoma is considerably lower^24^), and constructed rural evaluation units (EUs) of approximately 200,000–300,000 people, as a compromise between the WHO-recommended^25^ 100,000–250,000-person EU size, and the relatively low perceived likelihood of identifying populations here in which trachoma was a public health problem. (A similar compromise has been reached elsewhere.^26,27^) This produced a total of 43 EUs (Table 1). In each of these, a separate EU-level survey was planned and commenced. (In one EU, however, insecurity prevented completion.)

**Table 1.** Districts in which baseline trachoma prevalence surveys were commenced, Global Trachoma Mapping Project, Pakistan, October–December 2015.

Province	#	District	Population	Number of evaluation units
Baluchistan	1	Nasirabad	314,744	1
	2	Nushki	176,000	1
Khyber Pakhtunkhwa	3	Karak	551,419	2
	4	Mansehra	1,475,634	5
	5	Mardan	1,868,928	4
Punjab	6	Gujranwala	4,353,203	6
	7	Layyah	1,434,817	4
	8	Lodhran	1,499,904	4
	9	Sahiwal	2,359,288	5
Sindh	9	Ghotki	1,242,303	3
	10	Jamshoro	726,642	2^a^
Azad Jammu & Kashmir	12	Bagh	341,254	1
	13	Kotli	954,880	3
Gilgit-Baltistan	14	Ghizer	153,879	1
	15	Ghanche	113,108	1
	Total		17,566,004	43

^a^Completion of mapping was not possible in one of the two evaluation units of Jamshoro.

Following standard GTMP approaches,^28,29^ a two-stage cluster sampling method was used to determine which villages and households would be included in each survey. In each EU, 26 villages were randomly selected. Within each of those villages, a compact segment of 30 households was randomly selected and residents of those 30 households aged ≥1 year were enumerated and invited to participate.

### Field team structure and training

In Baluchistan and Khyber Pakhtunkhwa, each survey team was composed of one female grader, one male grader, one female recorder and one male recorder. In these provinces, for cultural reasons, female and male team members were unable to access male and female sections of the household, respectively, or examine household residents of the opposite gender. Each team had a driver and was accompanied by a local village guide. In Sindh, Punjab, Azad Jammu & Kashmir, and Gilgit-Baltistan, each survey team was composed of one grader and one recorder, with one driver for every 1–2 teams, depending on the geography of deployment; as in Baluchistan and Khyber Pakhtunkhwa, each team was accompanied by a local village guide. Ophthalmologists supervised the teams.

Extensive searches around Faisalabad for trachoma-endemic localities in which to undertake field-based grader training and testing resulted in very few cases of active trachoma being identified, and it was resolved instead to undertake this process in Sokoto, Nigeria, where a number of local government areas still had TF prevalences of 5.0–9.9%.^30^ In August 2015, a total of 23 grader trainees went to Sokoto; 16 passed the inter-grader agreement tests and were certified to participate in the surveys.^28^ Three recorder trainers were also trained in Sokoto; they later conducted workshops in Pakistan to train 26 recorders. Version 2 of the GTMP training package^31^ was used. This included the use of questions about previous trichiasis management being asked of survey subjects found to have trichiasis,^32^ but not recording of the presence or absence of trachomatous conjunctival scarring (TS)^9^ in eyes diagnosed with trichiasis.^33^ The latter element (recording of the presence or absence of TS) was introduced half-way through the surveys, in the form of Version 3 of the GTMP system; this was introduced to field team members via a specific training session.^34^ TS data are therefore only available for the last 19 EUs mapped.

### Fieldwork

Field teams liaised with district health authorities to find selected villages. Teams then created maps to plan survey routes. Schedules were communicated to the programme officer and supervisors on a weekly basis, to facilitate support.

Once in the field, given potential security concerns, particular emphasis was given to gaining district-, community-, household- and individual-level consent for the survey. The ophthalmologist led community-level engagement. The voluntary nature of participation by community residents was emphasized to communities throughout the survey process. Explicit consent from heads of households was required before field teams entered any household. Official government mission documents, identity cards, up-to-date points of contact and escalation channels were carried by field teams at all times.

All adults and children aged ≥1 year and residents in selected households were registered and asked to consent to eye examination. Grading was undertaken under 2.5× magnification, and the presence or absence of trichiasis, TF and TI recorded, using standard WHO simplified trachoma grading system definitions.^9^ When an eye was diagnosed as having trichiasis, the subject was asked whether a health worker had previously recommended surgery or epilation; for (only) the last 19 EUs, in addition, the presence or absence of TS in that eye was recorded.

Data were collected electronically using LINKS/GTMP software based on Open Data Kit, running on the Android operating system.^28,35^ Teams moved from one household to the next. Data collected in the field were encrypted and securely transferred to a central data hosting, management, approval, and reporting system, where they were rapidly analysed, as previously described.^28^

Graders used disposable latex gloves or cleaned their hands with 70% alcohol after each examination to avoid spreading infection. Basic treatment, such as 1% tetracycline eye ointment or analgesics, were provided on the spot to those in need. Patients requiring referral to hospital, including those with trichiasis, were promptly referred.

### Analysis

Standard GTMP approaches^28,29^ were used for data cleaning, analysis, health ministry review, and approval. In the first 23 completed EUs, lacking information on the presence or absence of TS in eyes with trichiasis, we calculated the prevalence of “any trichiasis”; for the last 19 completed EUs, we calculated the prevalence of both any trichiasis and “trichiasis+TS”, defined here as the presence of both trichiasis and TS (or the inability of the examiner to evert the lid to look for TS, presumed to be due to dense scarring) in the same eye.

### Ethical considerations

Ethical approval was given by the National Committee of Eye Health (1349/15) and the ethics committee of the London School of Hygiene & Tropical Medicine (6319 and 8355). For many districts, non-objection certificates were also obtained from the relevant District Health Authority. Because of low levels of literacy in the target population, field teams obtained consent from prospective participants verbally. Consent for individuals aged <15 years was given by a parent or guardian. Individuals with trachoma or other medical problems that were diagnosed by the field team were treated or referred (using a formal, specifically designed referral slip), as appropriate.

## Results

Fieldwork was undertaken from October to December 2015. Insecurity prevented completion of mapping in one EU; the incomplete data have been excluded from this presentation. The mean number of children examined per household varied considerably from one EU to another. In some EUs, recruitment of children in the initially-selected 26 villages yielded low numbers of 1–9-year-olds examined: in one case, this number was 558. Extra villages were selected (at random, using the original list of villages in the EU) in these EUs to compensate for the unexpectedly small household size. In some EUs, when mean household-level GPS co-ordinates were plotted by village, there was a clear overlap between the previously planned sub-Tehsil-level EUs. Borders for each EU occasionally needed to be adjusted for survey purposes (with the creation of new shapefiles), resulting in uneven numbers of villages sampled (Table 2).

**Table 2.** Number of 1–9-year-olds and number of ≥15-year-olds resident, examined, absent and refused; prevalence of trachomatous inflammation—follicular (TF), and prevalence of trichiasis (and, where possible, trichiasis+TS, see text) unknown to the health system, by evaluation unit, Global Trachoma Mapping Project, Pakistan, October–December 2015.

			1–9-year-olds						≥15-year-olds						
District	Evaluation unit	Number of villages sampled	Resident	Examined	Absent	Refused	Other	TF prevalence^a^, % (95% CI)	Resident	Examined	Absent	Refused	Other	Trichiasis prevalence^b^, % (95% CI)	Trichiasis+TS prevalence^b^, %(95% CI)
Nasirabad	Chattar Sub-Tehsil, Dera Murad Jamali Tehsil	26	2228	2227	1	0	0	1.3 (0.6–2.2)	1960	1889	62	9	0	0.01 (0.0–0.02)	0.01 (0.0–0.02)
Nushki	Nushki Tehsil	26	2119	2103	10	6	0	0.2 (0.0–0.4)	2325	2207	69	49	0	0.0 (0.0–0.0)	0.0 (0.0–0.0)
Karak	Banda Daud Shah Tehsil, Karak Tehsil	37	2096	1894	199	2	1	9.1 (6.6–11.6)	3981	3002	814	159	6	0.13 (0.0–0.24)	ND
Karak	Takhat Nasrati Tehsil	21	1431	1245	184	1	1	9.4 (5.6–13.0)	2038	1538	439	61	0	0.0 (0.0–0.0)	ND
Mansehra	Bala Kot Tehsil	26	1425	1272	152	1	0	1.0 (0.3–1.6)	2037	1598	372	67	0	0.0 (0.0–0.0)	ND
Mansehra	FR Kala Dhaka	26	1490	1437	52	1	0	13.6 (9.3–18.3)	1892	1526	341	25	0	0.0 (0.0–0.0)	ND
Mansehra	Mansehra Tehsil 1	27	1439	1281	151	7	0	2.0 (1.0–3.3)	2159	1585	439	135	0	0.0 (0.0–0.0)	ND
Mansehra	Mansehra Tehsil 2	26	1190	1067	123	0	0	4.1 (2.0–6.6)	2141	1674	442	25	0	0.0 (0.0–0.0)	ND
Mansehra	Oghi Tehsil	26	1221	1160	61	0	0	6.0 (4.0–7.6)	2142	1657	418	67	0	0.0 (0.0–0.0)	ND
Mardan	Mardan Tehsil 1	27	1569	1530	39	0	0	5.7 (4.0–7.4)	2057	1606	419	32	0	0.0 (0.0–0.0)	ND
Mardan	Mardan Tehsil 2	35	1974	1928	46	0	0	5.6 (4.0–7.2)	2745	2188	514	43	0	0.0 (0.0–0.0)	ND
Mardan	Mardan Tehsil 3	16	877	851	26	0	0	3.9 (2.0–5.6)	1253	1018	222	13	0	0.0 (0.0–0.0)	ND
Mardan	Takht Bhai Tehsil	27	1533	1506	26	1	0	3.7 (2.3–5.1)	2155	1724	396	35	0	0.0 (0.0–0.0)	ND
Gujranwala	Gujranwala Tehsil 1	30	960	952	0	7	1	1.0 (0.1–2.2)	2491	2396	11	82	2	0.02 (0.0–0.07)	ND
Gujranwala	Gujranwala Tehsil 2	22	784	784	0	0	0	0.2 (0.0–0.5)	2047	2042	0	5	0	0.06 (0.0–0.15)	ND
Gujranwala	Kamoke Tehsil	26	1184	1023	19	142	0	0.3 (0.0–0.6)	2210	1932	161	117	0	0.03 (0.0–0.09)	ND
Gujranwala	Nowshera Tehsil	26	1718	1713	1	4	0	0.9 (0.0–1.6)	1938	1920	11	4	3	0.03 (0.0–0.08)	ND
Gujranwala	Wazirabad Tehsil 1	29	929	878	39	12	0	0.9 (0.1–2.3)	2404	2173	171	60	0	2.96 (0.71–5.76)	ND
Gujranwala	Wazirabad Tehsil 2	23	964	952	12	0	0	1.6 (0.6–3.0)	2002	1886	96	20	0	0.07 (0.0–0.20)	0.0 (0.0–0.0)
Layyah	Choubara Tehsil	38	1227	1220	6	1	0	3.4 (2.0–4.7)	2896	2709	57	130	0	0.0 (0.0–0.0)	ND
Layyah	Karor Tehsil	26	899	896	2	1	0	0.9 (0.2–2.0)	1761	1593	6	162	0	0.08 (0.0–0.25)	0.08 (0.0–0.25)
Layyah	Leiah Tehsil 1	26	1092	1083	7	2	0	0.8 (0.0–2.0)	2313	2276	30	7	0	0.0 (0.0–0.0)	0.0 (0.0–0.0)
Layyah	Leiah Tehsil 2	26	1129	1121	5	3	0	0.2 (0.0–0.6)	2143	2107	34	2	0	0.01 (0.0–0.04)	0.0 (0.0–0.0)
Lodhran	Dunyapur Tehsil	26	1315	1256	3	56	0	1.8 (1.1–2.6)	2274	1963	206	105	0	0.0 (0.0–0.0)	ND
Lodhran	Kahror Tehsil	25	1405	1286	119	0	0	2.5 (1.0–3.7)	2479	2274	193	12	0	0.0 (0.0–0.0)	ND
Lodhran	Lodhran Tehsil 1	25	1743	1743	0	0	0	1.2 (0.4–2.3)	2391	2369	20	2	0	0.02 (0.0–0.06)	0.0 (0.0–0.0)
Lodhran	Lodhran Tehsil 2	27	2244	2243	1	0	0	1.8 (0.9–3.1)	2162	2132	27	3	0	0.08 (0.0–0.16)	0.08 (0.0–0.16)
Sahiwal	Chichawatni Tehsil 1	26	1196	1117	44	34	1	0.6 (0.2–1.2)	2342	1992	257	93	0	0.02 (0.0–0.07)	0.0 (0.0–0.0)
Sahiwal	Chichawatni Tehsil 2	26	829	768	56	5	0	0.9 (0.0–2.4)	2798	2235	554	9	0	0.02 (0.0–0.6)	0.02 (0.0–0.06)
Sahiwal	Sahiwal Tehsil 1	27	1088	988	90	10	0	2.3 (1.2–4.0)	2324	2279	42	3	0	0.0 (0.0–0.0)	0.0 (0.0–0.0)
Sahiwal	Sahiwal Tehsil 2	26	935	906	29	0	0	1.4 (0.7–2.3)	2588	2510	50	28	0	0.0 (0.0–0.0)	0.0 (0.0–0.0)
Sahiwal	Sahiwal Tehsil 3	33	1175	1157	6	12	0	0.3 (0.0–0.8)	2570	2449	102	17	2	0.0 (0.0–0.0)	ND
Ghotki	Dahrki Taluka	26	1268	1261	7	0	0	0.7 (0.0–1.8)	1865	1839	26	0	0	0.0 (0.0–0.0)	0.0 (0.0–0.0)
Ghotki	Ghotki Taluka, Khangarh Taluka	27	1013	1010	3	0	0	0.5 (0.1–0.8)	2112	2030	81	1	0	0.36 (0.14–0.67)	0.14 (0.0–0.34)
Ghotki	Mirpur Mathelo Taluka, Ubauro Taluka	26	903	895	8	0	0	0.8 (0.3–1.4)	2106	2024	81	1	0	0.03 (0.0–0.09)	0.03 (0.0–0.09)
Jamshoro	Sehwan Taluka	26	994	993	1	0	0	0.1 (0.0–0.4)	1767	1757	10	0	0	0.0 (0.0–0.0)	ND
Bagh	Dhirkot, Bagh, Hari Ghal	26	1269	1206	63	0	0	0.0 (0.0–0.0)	2749	2508	234	7	0	0.0 (0.0–0.0)	0.0 (0.0–0.0)
Kotli	Kotli Sub–Tehsil 1	26	1770	1730	32	8	0	0.1 (0.0–0.3)	2456	2113	320	23	0	0.0 (0.0–0.0)	ND
Kotli	Kotli Sub–Tehsil 2	27	1915	1868	33	14	0	0.2 (0.0–0.4)	2427	2082	313	32	0	0.14 (0.06–0.25)	0.13 (0.05–0.24)
Kotli	Kotli Sub–Tehsil 3	24	1393	1382	3	8	0	0.1 (0.0–0.3)	2067	1980	86	1	0	0.49 (0.22–0.87)	0.45 (0.19–0.82)
Ghizer	Ghizer	26	1198	1193	5	0	0	0.5 (0.1–1.3)	2897	2600	295	2	0	0.14 (0.06–0.25)	0.13 (0.05–0.24)
Ghanche	Ghanche	26	1214	1209	5	0	0	0.6 (0.0–1.6)	2804	2450	354	0	0	0.49 (0.22–0.87)	0.45 (0.19–0.82)

^a^Adjusted for age in 1-year age bands (see text)

^b^Unknown to the health system, adjusted for gender and age in 5-year age bands (see text). CI, confidence interval; ND, no data

In all, a total of 33,432 households were visited in 1119 clusters across 42 EUs. Those households were home to 56,345 1–9-year-olds and 96,268 ≥ 15-year-olds.

**Figure 1.**
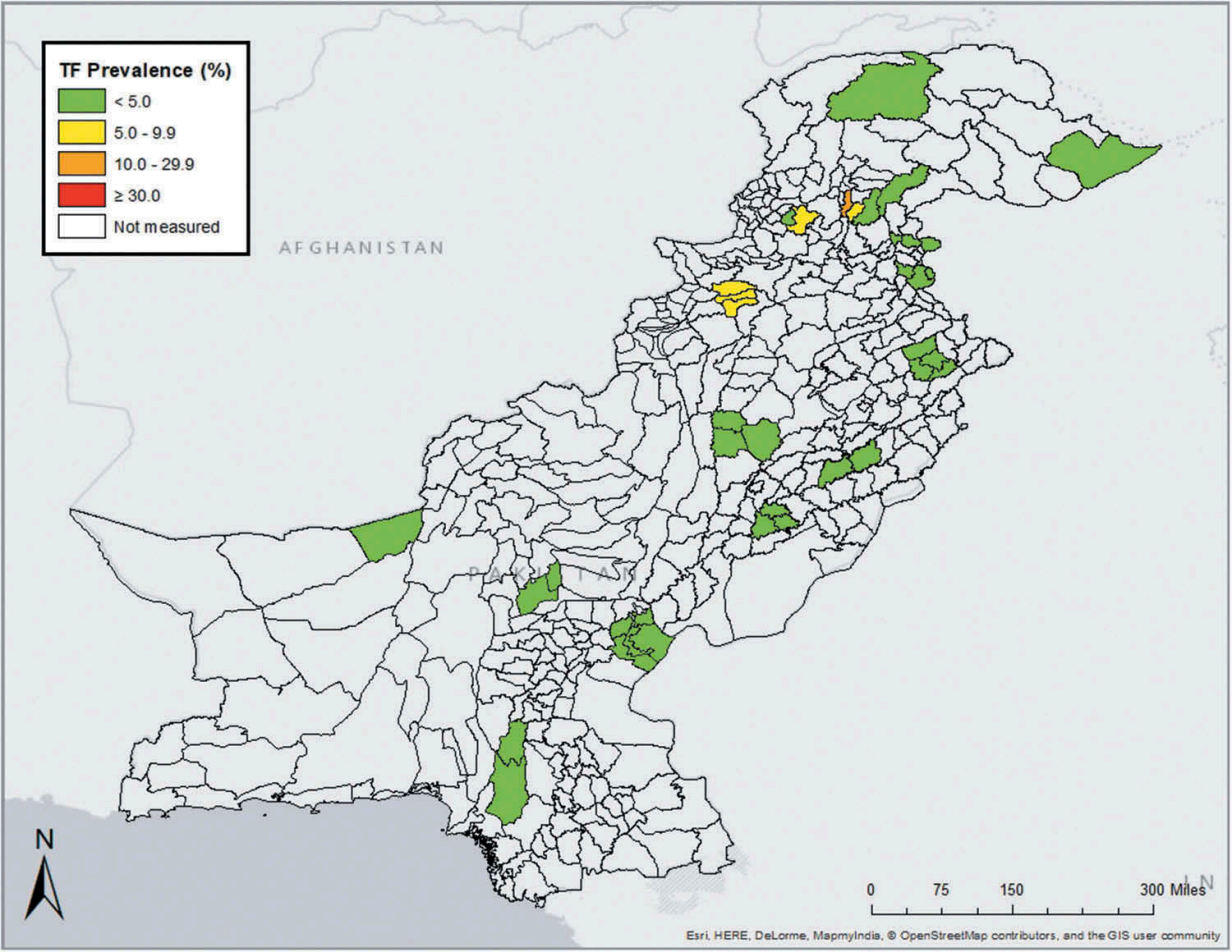
Evaluation-unit-level prevalence of trachomatous inflammation—follicular (TF), adjusted for age (see text), Global Trachoma Mapping Project, Pakistan, October–December 2015.

Of the 56,345 enumerated 1–9-year-olds, 54,334 (96%) consented and were examined, 1669 (3%) were absent on the day of the visit, 338 (1%) refused consent and 4 were not examined for other reasons. A mean of 1294 1–9-year-olds was examined per EU (range 768–2243). As shown in Table 2, the age-adjusted EU-level TF prevalence in 1–9-year-olds ranged from 0% in the EU covering Dhirkot, Bagh and Hari Ghal, to 13.6% (95%CI 9.3–18.3) in FR Kala Dhaka. Six (14%) of 42 EUs had age-adjusted TF prevalence estimates of ≥5% (Figure 1, Table 2).

**Figure 2.**
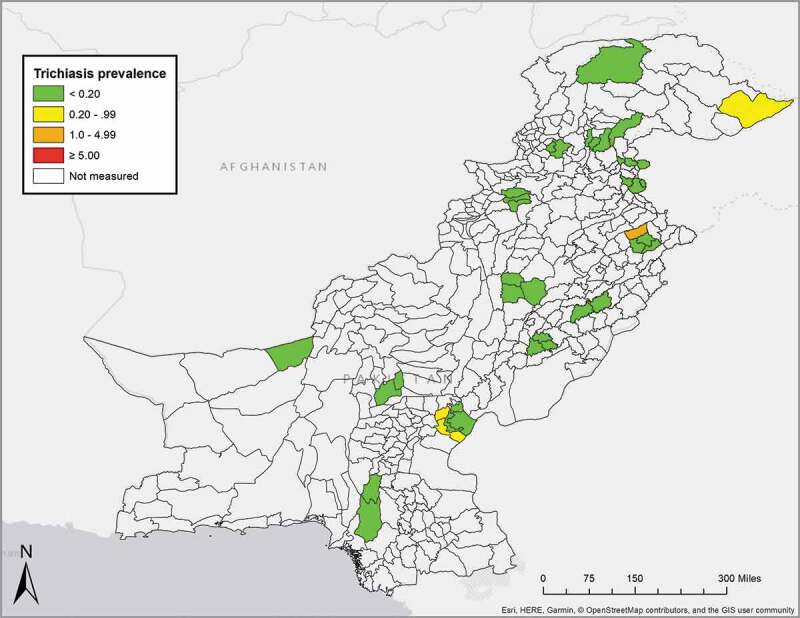
Evaluation unit (EU)-level prevalence of trichiasis unknown to the health system in ≥15-year-olds, adjusted for gender and age (see text), Global Trachoma Mapping Project, Pakistan, October–December 2015.

Of 96,268 enumerated ≥15-year-olds, 85,832 (89%) were examined, 8775 (9%) were absent on the day of the visit, 1648 (2%) did not agree to participate and 13 were not examined for other reasons (Table 2). The age- and gender-adjusted trichiasis prevalence in ≥15-year-olds (regardless of the presence or absence of TS) ranged from 0% to 2.96%. Twenty-two (52%) of 42 EUs had age- and gender-adjusted trichiasis prevalence estimates of 0%, 16 (38%) had non-zero trichiasis prevalence estimates that were less than the elimination threshold of 0.2%,^17^ and four (10%) had trichiasis prevalence estimates of ≥0.2% in adults (Figure 2, Table 2). In only one EU (Ghotki Taluka, Khangarh Taluka) was the adjusted prevalence of trichiasis+TS substantially different from the adjusted prevalence of trichiasis (Table 2).

Household-level access to an improved source of water for face and hand washing ranged from 37% in the EU covering Chattar Sub-Tehsil and Dera Murad Jamali Tehsil, to 100% in 22 (52%) of the 42 EUs surveyed. In 36 (86% of) EUs, ≥80% of households had access to an improved source of water for face and hand washing (Table 3).

**Table 3.** Household-level access to water and sanitation, by evaluation unit, Global Trachoma Mapping Project, Pakistan, October–December 2015.

Evaluation unit (* asterisked evaluation units are those in which the estimated TF prevalence was ≥5%)	Households, n	Improved washing water, n (%)	Washing water <1km, n (%)	Improved latrine, n (%)
Chattar Sub-Tehsil, Dera Murad Jamali Tehsil	780	290 (37)	740 (95)	214 (27)
Nushki Tehsil	780	602 (77)	571 (73)	445 (57)
Banda Daud Shah Tehsil, Karak Tehsil*	1119	979 (87)	1023 (91)	895 (80)
Takhat Nasrati Tehsil*	633	559 (88)	560 (88)	430 (68)
Bala Kot Tehsil	781	770 (99)	770 (99)	759 (97)
FR Kala Dhaka*	781	563 (72)	672 (86)	406 (52)
Mansehra Tehsil 1	792	718 (91)	790 (100)	741 (94)
Mansehra Tehsil 2	782	767 (98)	743 (95)	736 (94)
Oghi Tehsil*	785	742 (95)	739 (94)	707 (90)
Mardan Tehsil 1*	812	811 (100)	812 (100)	693 (85)
Mardan Tehsil 2*	1024	937 (92)	1018 (99)	801 (78)
Mardan Tehsil 3	511	504 (99)	511 (100)	430 (84)
Takht Bhai Tehsil	812	811 (100)	811 (100)	620 (76)
Gujranwala Tehsil 1	890	889 (100)	890 (100)	887 (100)
Gujranwala Tehsil 2	654	654 (100)	654 (100)	654 (100)
Kamoke Tehsil	758	758 (100)	758 (100)	749 (99)
Nowshera Tehsil	796	796 (100)	796 (100)	794 (100)
Wazirabad Tehsil 1	850	847 (100)	844 (99)	828 (97)
Wazirabad Tehsil 2	687	687 (100)	687 (100)	668 (97)
Choubara Tehsil	1126	1122 (100)	1126 (100)	583 (52)
Karor Tehsil	761	761 (100)	761 (100)	728 (96)
Leiah Tehsil 1	814	814 (100)	814 (100)	771 (95)
Leiah Tehsil 2	795	795 (100)	795 (100)	641 (81)
Dunyapur Tehsil	757	747 (99)	752 (99)	653 (86)
Kahror Tehsil	712	712 (100)	712 (100)	415 (58)
Lodhran Tehsil 1	758	758 (100)	757 (100)	426 (56)
Lodhran Tehsil 2	819	758 (93)	780 (95)	658 (80)
Chichawatni Tehsil 1	750	750 (100)	750 (100)	663 (88)
Chichawatni Tehsil 2	761	760 (100)	760 (100)	641 (84)
Sahiwal Tehsil 1	814	811 (100)	784 (96)	695 (85)
Sahiwal Tehsil 2	799	795 (99)	701 (88)	620 (78)
Sahiwal Tehsil 3	994	993 (100)	983 (99)	927 (93)
Dahrki Taluka	780	777 (100)	780 (100)	779 (100)
Ghotki Taluka, Khangarh Taluka	809	809 (100)	809 (100)	492 (61)
Mirpur Mathelo Taluka, Ubauro Taluka	787	787 (100)	712 (90)	133 (17)
Sehwan Taluka	784	473 (60)	444 (57)	359 (46)
Dhirkot, Bagh, Hari Ghal	752	744 (99)	741 (99)	745 (99)
Kotli Sub-Tehsil 1	760	707 (93)	740 (97)	746 (98)
Kotli Sub-Tehsil 2	795	795 (100)	765 (96)	772 (97)
Kotli Sub-Tehsil 3	731	313 (43)	649 (89)	657 (90)
Ghizer	780	656 (84)	755 (97)	694 (89)
Ghanche	767	594 (77)	712 (93)	48 (6)

The proportion of households with access to an (improved or unimproved) source of water for face and hand washing within 1 km of the house ranged from 57% in the EU covering Sehwan Taluka to 100% in 19 (45%) of the 42 EUs surveyed. In 40 (95%) of the 42 EUs surveyed, ≥80% of households had access to a source of water for face and hand washing within 1 km of the house (Table 3).

Household-level access to improved sanitation ranged from a low of 6% in Genchi to a high of 100% in 4 (10%) of 42 EUs. In 23 (55%) of the EUs, ≥80% of households had access to improved sanitation (Table 3).

## Discussion

Data from this fourth phase of baseline mapping represent another important step towards complete categorization of the distribution and burden of trachoma in Pakistan. Only three of 42 EUs mapped were shown to qualify for public-health-level deployment of trichiasis surgical services, and only six EUs (all in Khyber Pakhtunkhwa) were shown to qualify for intervention with A, F, and E components of SAFE.^36^ There was no overlap between the group of three EUs qualifying for S and the group of six EUs qualifying for A, F, and E. The very high prevalence of trichiasis in Wazirabad Tehsil 1 (2.96%, 95%CI 0.71–5.76) was unexpected and therefore requires confirmation, likely through a repeat survey. Low prevalences of active trachoma in the 19 EUs we surveyed in Punjab match the low prevalence of active disease recorded in children of Dera Ghazi Khan district, Punjab, by other investigators, in 2014.^37^

Experience elsewhere suggests that SAFE strategy implementation would have a good chance of successfully eliminating trachoma here.^38–40^ Unfortunately, the recent outbreak of extensively drug-resistant typhoid in Pakistan^41,42^ may prevent the national trachoma programme from using azithromycin mass drug administration, the SAFE strategy intervention with the strongest evidence for effect.^43^ For EUs with TF prevalence estimates ≥5%, use of topical tetracycline eye ointment might be an option, though compliance with the prolonged course of treatment required could be anticipated to be low^44^, particularly amongst the large numbers of asymptomatic and minimally symptomatic individuals who would be offered it in a mass treatment campaign. Intensive implementation of the F and E components of the SAFE strategy^45,46^ could be another solution. We note that the prevalence of household-level access to water and sanitation varied markedly between EUs (Table 3), though several of the EUs with TF prevalence ≥5% already had levels of access to these services that associate with lower risk of (and even, for sanitation coverage ≥80%, “herd protection” against) active trachoma.^47,48^ We also note that the evidence base for the F and E interventions is weak.^49–51^

Not all trichiasis is caused by trachoma.^52^ Pakistan was one of the first GTMP deployments in which we were able to explore the effect of separating out trichiasis seen in association with TS from trichiasis noted in its absence. Guided by the Second Global Scientific Meeting on Trachomatous Trichiasis,^33^ half-way through this series of surveys, we asked field teams to start recording whether or not scarring was clearly visible in the conjunctivae of eyes with trichiasis; where they were unable to evert the eyelid to check, TS was assumed to be present. In this low-prevalence setting, this would have made a difference to the categorization of EUs as requiring or not requiring public health-level “S” interventions in only one instance. Further work to better understand the epidemiology, phenotypes and vision-threatening potential of trichiasis with and without visible scar is needed.^53^

Building on results from the GTMP in Pakistan and elsewhere,^54^ momentum towards global elimination of trachoma is progressively building.^55–57^ In April 2018, in London, Commonwealth Heads of Government pledged their commitment to the global target^58^ and the United Kingdom re-affirmed financial support.^59^ It is recommended that stakeholders in Pakistan take advantage of these developments, rapidly complete planning using the data presented here, and implement a national programme that can bring about trachoma elimination nationwide.

## Appendix

The Global Trachoma Mapping Project Investigators are: Agatha Aboe (1,11), Liknaw Adamu (4), Wondu Alemayehu (4,5), Menbere Alemu (4), Neal D. E. Alexander (9), Ana Bakhtiari (2,9), Berhanu Bero (4), Sarah Bovill (8), Simon J. Brooker (1,6), Simon Bush (7,8), Brian K. Chu (2,9), Paul Courtright (1,3,4,7,11), Michael Dejene (3), Paul M. Emerson (1,6,7), Rebecca M. Flueckiger (2), Allen Foster (1,7), Solomon Gadisa (4), Katherine Gass (6,9), Teshome Gebre (4), Zelalem Habtamu (4), Danny Haddad (1,6,7,8), Erik Harvey (1,6,10), Dominic Haslam (8), Khumbo Kalua (5), Amir B. Kello (4,5), Jonathan D. King (6,10,11), Richard Le Mesurier (4,7), Susan Lewallen (4,11), Thomas M. Lietman (10), Chad MacArthur (6,11), Colin Macleod (3,9), Silvio P. Mariotti (7,11), Anna Massey (8), Els Mathieu (6,11), Siobhain McCullagh (8), Addis Mekasha (4), Tom Millar (4,8), Caleb Mpyet (3,5), Beatriz Muñoz (6,9), Jeremiah Ngondi (1,3,6,11), Stephanie Ogden (6), Alex Pavluck (2,4,10), Joseph Pearce (10), Serge Resnikoff (1), Virginia Sarah (4), Boubacar Sarr (5), Alemayehu Sisay (4), Jennifer L. Smith (11), Anthony W. Solomon (1,2,3,4,5,6,7,8,9,10,11), Jo Thomson (4); Sheila K. West (1,10,11), Rebecca Willis (2,9).

1. Advisory Committee, 2. Information Technology, Geographical Information Systems, and Data Processing, 3. Epidemiological Support, 4. Ethiopia Pilot Team, 5. Master Grader Trainers, 6. Methodologies Working Group, 7. Prioritisation Working Group, 8. Proposal Development, Finances and Logistics, 9. Statistics and Data Analysis, 10. Tools Working Group, 11. Training Working Group.
